# Characterization of *Sinorhizobium* sp. LM21 Prophages and Virus-Encoded DNA Methyltransferases in the Light of Comparative Genomic Analyses of the Sinorhizobial Virome

**DOI:** 10.3390/v9070161

**Published:** 2017-06-26

**Authors:** Przemyslaw Decewicz, Monika Radlinska, Lukasz Dziewit

**Affiliations:** 1 Department of Bacterial Genetics, Institute of Microbiology, Faculty of Biology, University of Warsaw, Miecznikowa 1, 02-096 Warsaw, Poland; decewicz@biol.uw.edu.pl; 2 Department of Virology, Institute of Microbiology, Faculty of Biology, University of Warsaw, Miecznikowa 1, 02-096 Warsaw, Poland; m.radlinska@biol.uw.edu.pl

**Keywords:** *Sinorhizobium* sp. LM21, *Alphaproteobacteria*, prophage, DNA methyltransferase, CcrM-like specificity

## Abstract

The genus *Sinorhizobium/Ensifer* mostly groups nitrogen-fixing bacteria that create root or stem nodules on leguminous plants and transform atmospheric nitrogen into ammonia, which improves the productivity of the plants. Although these biotechnologically-important bacteria are commonly found in various soil environments, little is known about their phages. In this study, the genome of *Sinorhizobium* sp. LM21 isolated from a heavy-metal-contaminated copper mine in Poland was investigated for the presence of prophages and DNA methyltransferase-encoding genes. In addition to the previously identified temperate phage, ΦLM21, and the phage-plasmid, pLM21S1, the analysis revealed the presence of three prophage regions. Moreover, four novel phage-encoded DNA methyltransferase (MTase) genes were identified and the enzymes were characterized. It was shown that two of the identified viral MTases methylated the same target sequence (GANTC) as cell cycle-regulated methyltransferase (CcrM) of the bacterial host strain, LM21. This discovery was recognized as an example of the evolutionary convergence between enzymes of sinorhizobial viruses and their host, which may play an important role in virus cycle. In the last part of the study, thorough comparative analyses of 31 sinorhizobial (pro)phages (including active sinorhizobial phages and novel putative prophages retrieved and manually re-annotated from *Sinorhizobium* spp. genomes) were performed. The networking analysis revealed the presence of highly conserved proteins (e.g., holins and endolysins) and a high diversity of viral integrases. The analysis also revealed a large number of viral DNA MTases, whose genes were frequently located within the predicted replication modules of analyzed prophages, which may suggest their important regulatory role. Summarizing, complex analysis of the phage protein similarity network enabled a new insight into overall sinorhizobial virome diversity.

## 1. Introduction

*Alphaproteobacteria* constitute a physiologically diverse group of bacteria, whose representatives were isolated from various environments and exhibit diverse metabolic properties. Amongst *Alphaproteobacteria*, there are symbiotic, nitrogen-fixing bacteria (e.g., *Rhizobium* spp. and *Sinorhizobium* spp.) [1], methylotrophs utilizing one-carbon compounds (e.g., *Paracoccus* spp.) [2], and obligate intracellular parasites (e.g., *Rickettsia* spp.) [3]. Currently (as of 1 March 2017), according to the National Centre for Biotechnology Information (NCBI) genome browser, full genomic sequences of 527 representatives of *Alphaproteobacteria* are available. Although much is known about the metabolic properties of *Alphaproteobacteria* and their genomes’ architecture and functioning [4,5,6,7,8,9], surprisingly, up till now, less than 90 phages infecting this class of bacteria have been identified.

The genus *Sinorhizobium* mostly groups nitrogen-fixing bacteria, creating root or stem nodules on leguminous plants. Sinorhizobia can transform N_2_ into ammonia, which improves the overall productivity of the plants [9,10,11]. Interestingly, to our knowledge, only eight active phages of *Sinorhizobium* spp. have been identified and described so far, including five lytic phages (ΦM12 (GenBank accession No. KF381361), ΦM7 (GenBank accession No. KR052480), ΦM19 (GenBank accession No. KR052481), ΦM9 (GenBank accession No. KP881232), and ΦN3 (GenBank accession No. KR052482)) [12,13], and three temperate viruses (Φ16-3 (GenBank accession No. DQ500118), ΦPBC5 (GenBank accession No. AF448724), and ΦLM21 (GenBank accession No. KJ743987)) [14,15].

*Sinorhizobium* sp. LM21 was isolated from the heavy-metal-contaminated copper mine located in the Lubin-Glogow Copper District in Lower Silesia Province (Poland). The presence of heavy metals in this environment may explain the hyper-tolerance of the LM21 strain to As^3+^ (the minimal inhibitory concentration (MIC) is 5 mM), As^5+^ (200 mM), Cd^2+^ (2 mM), Co^2+^ (1.5 mM), Cu^2+^ (5 mM), Ni^2+^ (4 mM), and Zn^2+^ (3 mM). Moreover, the strain utilizes several polycyclic hydrocarbons, including anthracene, ferrocene, phenanthrene, and pyrene [16].

Our previous analyses of *Sinorhizobium* sp. LM21 revealed that it carries an unusual, putative plasmid-like prophage, pLM21S1 (GenBank accession No. KM659098), and an inducible temperate phage, ΦLM21 (GenBank accession No. KJ743987) [14,16]. Molecular characterization of the phage ΦLM21 revealed that it encodes DNA methyltransferase (PhiLM21_p027), exhibiting GANTC (methylated nucleotide is underlined) specificity, the same as the host-encoded CcrM (for “cell cycle-regulated methyltransferase”), which is an orphan, essential for bacterium viability, regulatory DNA methyltransferase (MTase), widespread among members of *Alphaproteobacteria* (it was found in all *Alphaproteobacteria* except *Rickettsiales* and *Magnetococcales*) [17,18]. The PhiLM21_p027 and the host CcrM_LM21_ proteins do not share sequence similarities. This is an interesting example of the convergent evolution between the virus and its native host enzyme, regarding their sequence specificity [14]. In this work, we analyzed the genome of the LM21 strain for the presence of other prophages. We identified three novel phage regions, which were thoroughly analyzed and carefully investigated for the presence of genes encoding DNA MTases to check if other prophages of this strain (like ΦLM21 previously) also encode modifying enzymes exhibiting CcrM-like specificity. We asked whether the phenomenon of mimicking regulatory mechanisms of the host by the virus encoding its own CcrM-like MTase is common in the genus *Sinorhizobium*. We also looked for other potential phage DNA MTase specificities widespread in *Alphaproteobacteria*. In the course of this study, in addition to the previously characterized (cellular CcrM and ΦLM21 CcrM-like) MTases, four novel prophage-encoded MTases were found. We showed that two of them also exhibited CcrM-like specificity. In the last part of the study, we screened the NCBI database for complete *Sinorhizobium* genomes identifying and re-annotating putative prophage regions. This enabled performing thorough comparative analyses of sinorhizobial viruses, showing that they encode a large amount of DNA MTases, frequently localized within their predicted replication modules.

## 2. Materials and Methods

### 2.1. Bacterial Strains, Plasmids, Media, and Growth Conditions

The following strains were used in this study: *Escherichia coli* TOP10F′ (Invitrogen, Waltham, MA, USA), *E. coli* ER2566 (New England BioLabs, Ipswich, MA, USA), *E. coli* ER2929 Dam^−^ strain lysogenized with DE3 element [19] and *Sinorhizobium* sp. LM21 [16]. *Sinorhizobium* sp. LM21 was grown in tryptone-yeast extract (TY) medium [20] at 30 °C. *E. coli* strains were cultured under standard conditions in lysogeny broth (LB) medium at 37 °C. When required, media were supplemented with kanamycin (Km) at 50 μg mL^−1^. Plasmids pET28a and pET30a (Invitrogen, Waltham, MA, USA) were used as expression vectors.

### 2.2. DNA Sequencing

Genomic DNA of the LM21 strain was isolated using the CTAB/Lysozyme method [20]. An Illumina TruSeq library was constructed following manufacturer’s instructions and sequenced applying Illumina MiSeq instrument (using the v3 chemistry kit) (Illumina, San Diego, CA, USA). Raw reads were filtered for quality and assembled using Newbler version 3.0 software (Roche, Basel, Switzerland).

### 2.3. Bioinformatics

The LM21 draft genome was automatically annotated using RAST server [21,22]. The prophage sequences within the draft genome were identified using PhiSpy [23] and manual inspection. Then, the predicted prophage sequences were manually annotated using Clone Manager (Sci-Ed8) and Artemis software [24]. Similarity searches were performed using the BLAST program [25] provided by the NCBI, UniProt [26], and Pfam databases [27]. Putative tRNA genes were searched using the tRNAScan-SE [28] and ARAGORN programs [29]. Protein conserved domains and motifs were searched using MOTIF Search [30] and HHpred tools [31]. The MTase genes were tracked using the BLAST search with the REBASE [32] resources as a query, and the obtained results were manually verified. Phage taxonomy assignment was performed using VIRFAM [33] and BLAST searches of large terminase subunit and major capsid protein sequences of sinorhizobial phages against *Caudovirales* phages indicated in ICTV Master Species List 2016 v1.3 [34] (ictvonline.org). The visualization of the comparative genomic analyses results was performed with the application of Circoletto [35] and Gephi [36]. Similarity network was constructed based on all against all BLAST results with the application of our custom Python script. In the network each node represents a single protein and each edge reflects reciprocated sequence similarity between two proteins (above given thresholds).

### 2.4. Standard Molecular Biology Procedures

Standard DNA manipulations were carried out according to the protocols described by Sambrook and Russell [20]. PCR reactions were performed with Phusion High Fidelity DNA polymerase (Thermo Fisher Scientific, Waltham, MA, USA).

### 2.5. Cloning, Overexpression, Purification, and Testing of Putative DNA MTases Activities

The predicted DNA MTase genes identified within prophages Φ2LM21 and Φ3LM21 were amplified using specific oligonucleotide primers (Table S1). Then, the PCR products (after purification) were digested with appropriate enzymes and ligated with pET30a or pET28a vector cut with the same enzymes as the DNA of a relevant insert. Restriction enzymes used for cloning, vectors, and names of resulting recombinant plasmids are listed in Table S1. The recombinant enzymes were expressed in the *E. coli* ER2566. Protein expression and restriction enzyme digestion protection assay for revealing the sequence specificity of particular MTases was performed as previously described [37].

### 2.6. Cloning, Overexpression and Testing of Φ2LM21-Encoded Lytic Enzyme Activity

The DNA encoding putative lytic enzyme (Phi2LM21_p54) of the Φ2LM21 prophage was amplified by PCR using primers listed in Table S1. DNA product was cleaved with *Nde*I and *Xho*I and cloned into appropriate sites of digested pET30a plasmid, yielding pET-lyt. Plasmid pET-lyt was introduced into *E. coli* ER2566 and the resulting strain was inoculated and cultured in LB medium supplemented with glucose (final concentration of 1.0%) to an optical density (OD_600_) of 0.35. Then, the culture was centrifuged, resuspended in fresh LB medium and divided into two equal volumes—one supplemented with glucose and the other with IPTG (Isopropyl β-d-1-thiogalactopyranoside) to a final concentration of 1 mM. Growth of these two cultures was monitored by measuring the optical density.

### 2.7. Nucleotide Sequence Accession Number

The whole-genome shotgun project of *Sinorhizobium* sp. LM21 has been deposited in the NCBI GenBank database under the accession number SAMN06765771.

## 3. Results and Discussion

### 3.1. Identification and Classification of the Sinorhizobium sp. LM21 Prophages

**Figure 1:**
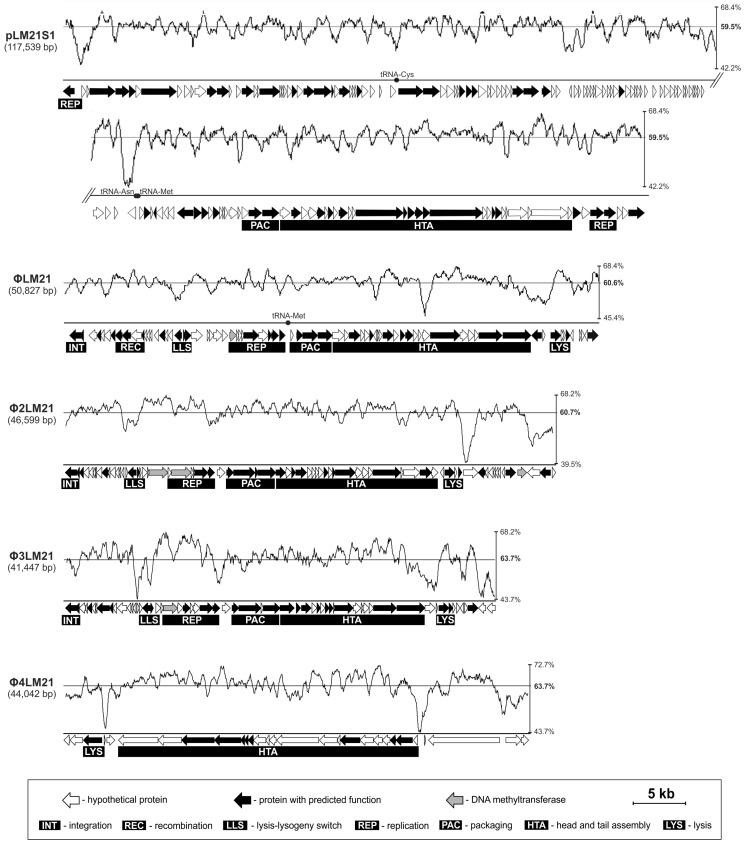
Genome organization of pLM21S1, ΦLM21, Φ2LM21 and Φ3LM21 prophages, and Φ4LM21 prophage remnant of *Sinorhizobium* sp. LM21. Arrows indicate the transcriptional orientation of the genes. The plots show the GC contents of the prophages. The genetic map of pLM21S1 was divided to retain transparency. The virus-specific genetic modules were indicated by the black boxes.

Only two *Sinorhizobium* sp. LM21 prophages have been identified thus far. These were plasmid-like prophage, pLM21S1, and temperate phage, ΦLM21 (Figure 1) [14,16]. The pLM21S1 (117.5 kb) is an unusual extrachromosomal element that carries a RepC-like replication system (typical for *repABC*-type plasmids of *Alphaproteobacteria* [38] and is related to phage RHEph10 (GenBank accession No. JX483881) of *Rhizobium etli* CFN42 [39]. It also carries genes encoding enzymes involved in nicotinamide adenine dinucleotide (NAD) biosynthesis [16]. The second identified virus was a temperate phage, ΦLM21 (50.8 kb) [14]. The ΦLM21 phage was identified as an active virus after the treatment of *Sinorhizobium* sp. LM21 cells with mitomycin C. It was the only phage that was induced with this method in *Sinorhizobium* sp. LM21, which may suggest that pLM21S1 and other putative prophages within the LM21 genome are inactive, or alternatively, they may require specific, as yet unidentified environmental factors for induction.

In the course of this study, the draft genome sequence of *Sinorhizobium* sp. LM21 was obtained. It was assembled into 136 contigs (the size ranged from 103 to 1,033,074 bp) with a total length of 7,615,909 bp and 62.26% GC content. Automatic annotation performed with the application of the RAST server revealed the presence of 7627 genes (including 55 tRNA genes). The total length of predicted genes was 6,673,962 bp, which comprises 87.9% of the genome.

Obtaining the LM21 draft genomic sequence enabled us to perform searches of other prophages. With the use of the PhiSpy tool and manual inspection, besides the abovementioned ΦLM21 and pLM21S1, we distinguished three novel prophage regions, which were named Φ2LM21, Φ3LM21, and Φ4LM21, respectively (Files S1–S3). Based on the predicted proteomes of the distinguished prophages, the VIRFAM tool [33] classified Φ2LM21 and Φ3LM21 into the *Siphoviridae*, and Φ4LM21 into the *Myoviridae* family.

### 3.2. Characterization of the Φ2LM21 and Φ3LM21 Prophages

Two of the DNA regions distinguished within the LM21 genome and containing clusters of viral genes most probably comprise complete prophages. Their predicted genome sizes are 46,599 bp for Φ2LM21 and 41,447 bp for Φ3LM21, and the GC content (60.65% and 61.24%, respectively) is slightly lower than the GC content of the LM21 genome (62.26%). For the Φ2LM21 and Φ3LM21 prophages, the manual annotation revealed the presence of 69 and 59 genes, respectively. The specific functions were assigned for 27 and 28 of those genes, respectively (Tables S2 and S3). The gene content and structural organization of functional Φ2LM21 and Φ3LM21 modules were similar to the previously described active phage ΦLM21, which was also classified into the *Siphoviridae* family.

The integration/excision modules of the Φ2LM21 and Φ3LM21 phages contain the tyrosine integrase genes (*phi2LM21_p01* and *phi3LM21_p01*, respectively). The Φ2LM21-encoded integrase (360 amino acids (aa)) exhibited the highest identity (98%), with the integrases widely distributed in many *Sinorhizobium/Ensifer* genomes (e.g., GenBank accession Nos. OCP05027 and OCP11814). The Φ3LM21-encoded integrase (371 aa) showed the highest identity (83%) with site-specific integrase/recombinase of *Rhizobium* phage vB_RleM PPF1 (GenBank accession No. YP_009099606). It is noteworthy that Phi2LM21_p01 and Phi3LM21_p01 proteins and also an integrase of the ΦLM21 phage (GenBank accession No. AII27753) do not show significant sequence similarity. It was also possible to distinguish the potential attachment sites (*attB*) for both prophages. Ф2LM21 and Ф3LM21 integrated into phenylalanine tRNA (tRNA-Phe (GAA)) and proline tRNA (tRNA-Pro (CGG)) genes, respectively. Downstream of the predicted prophage regions, sequences identical to the first 17 and 57 nucleotides of the Ф2LM21 and Ф3LM21 genomes, respectively, could be identified. It is noteworthy that the previously identified phage, ΦLM21, was integrated into another proline tRNA (tRNA-Pro (GGG)) gene, and, in all abovementioned cases, integration reconstituted an intact copy of the appropriate genes.

The lysogeny control region in Φ2LM21 and Φ3LM21 is composed of two genes, i.e., *phi2LM21_p16* and *phi2LM21_p17* in Φ2LM21, and *phi3LM21_p17* and *phi3LM21_p18* in Φ3LM21. The first gene in each pair encodes a CI repressor-like protein (as the HTH_CROC1 (Cro/C1-type helix-turn-helix) conserved domain was identified in it), which is leftward orientated. The subsequent gene in each prophage most probably encodes Cro-like protein (as predicted using MOTIF Search and HHpred tools), which is in rightward orientation (Figure 1).

We predicted that *phi2LM21_p26* and *phi3LM21_p26* encode putative replication initiation proteins of prophages Φ2LM21 and Φ3LM21, respectively. Both proteins are homologous (28% of reciprocal identity) and contain a helix-turn-helix domain, which is most probably responsible for their interactions with DNA. Interestingly, within the replication modules of both prophages, the DNA MTase genes were also identified including: (i) *phi2LM21_p23* (in Φ2LM21), encoding C5 cytosine-specific DNA methyltransferase (m^5^C MTase); and (ii) *phi2LM21_p21* (in Φ2LM21) and *phi3LM21_p21* (in Φ3LM21), encoding m^6^A MTases. We speculate that those enzymes may participate in regulation of phage replication. It is worth mentioning that, at the left arm of the prophage Φ2LM21, another m^6^A MTase gene (*phi2LM21_p66*) was found, which means that Φ2LM21 encodes, in total, three DNA MTases (Figure 1).

The packaging module is essential for packaging of virus DNA into the phage head [40]. In both prophages, Φ2LM21 and Φ3LM21, those modules are composed of two genes encoding small (*terS*) and large (*terL*) subunits of terminase, *phi2LM21_p29-30* and *phi3LM21_p29-30*, respectively (Figure 1). Phi2LM21_p30 and Phi3LM21_p30 proteins belong to the phage terminase large subunit (GpA) superfamily (COG5525) [40] and exhibit 99% identity to prophage large subunits of terminase of *Sinorhizobium* sp. NFACC03 (GenBank accession No. SDA39297) and 88% identity to TerL protein of *Ensifer* sp. Root142 (GenBank accession No. WP_057224692), respectively. Sequence similarity between Phi2LM21_p30 and Phi3LM21_p30 was very low (21%), while both enzymes did not show sequence similarity with the ΦLM21-encoded TerL protein (GenBank accession No. AII27790).

In both phages, adjacent to the packaging modules, the gene clusters (*phi2LM21_p31-52* and *phi3LM21_p32-48*, respectively) encoding phage structural proteins were identified. In Φ2LM21, the putative function could be assigned for 10 of them: (i) head-to-tail joining protein (Phi2LM21_p31); (ii) portal protein (Phi2LM21_p32); (iii) head maturation protease (Phi2LM21_p33); (iv) head decoration protein D (Phi2LM21_p35); (v) major capsid protein (Phi2LM21_p36); (vi) major tail protein (Phi2LM21_p41); (vii) tail assembly chaperone (Phi2LM21_p43); (viii) tail tape measure protein (Phi2LM21_p44); and (ix–x) two tail fiber proteins (Phi2LM21_p48 and Phi2LM21_p51). In Φ3LM21, the function could be assigned also for 10 predicted structural proteins and those were: (i) portal protein (Phi3LM21_p32); (ii) head maturation protease (Phi3LM21_p33); (iii) virion structural protein (Phi3LM21_p34); (iv) major capsid protein (Phi3LM21_p35); (v) head-to-tail joining protein (Phi3LM21_p37); (vi) major tail protein (Phi3LM21_p39); (vii) tail assembly chaperone (Phi3LM21_p40); (viii) tail tape measure protein (Phi3LM21_p42); (ix) tail protein (Phi3LM21_p47); and (x) tail fiber protein (Phi3LM21_p48) (Figure 1). The identified structural proteins share similarities with their functional counterparts of various phages and prophages of *Alphaproteobacteria*.

In both prophages, the host’s cell lysis genes are located downstream of the structural gene clusters. In Φ2LM21, those are *phi2LM21_p54* and *phi2LM21_p56*, encoding putative chitinase (COG3179) exhibiting 92% identity to chitinase of *Ensifer adhaerens* X097 (GenBank accession No. OKP79630), and holin belonging to holin superfamily III [41] with 98% identity to LydA phage holin of *Ensifer* sp. Root1298 (GenBank accession No. KQX55447) [42], respectively. To verify the function of Phi2LM21_p54 as a predicted lytic enzyme, we cloned its gene into the plasmid vector pET30a under the control of an inducible T7 promotor. It was shown that the induction of the *phi2LM21_p54* gene by IPTG had a lethal effect on the heterological host, resulting in cell lysis after 45 min (Figure S1). In Φ3LM21, only one gene (*phi3LM21_p51*) encoding lytic enzyme (putative chitinase (COG3179)) exhibiting the highest identity (83%) to several predicted chitinases of *Sinorhizobium meliloti* (GenBank accession Nos. WP_017267359, WP_027989971, andWP_028011802) was identified (Figure 1). This putative lytic enzyme is 82% identical with PhiLM21_p65 of ΦLM21, whose lytic activity was previously demonstrated experimentally [14].

In Φ2LM21 and Φ3LM21, the homologous genes (*phi2LM21_p65* and *phi3LM21B_p57*) encoding ATP-dependent DNA ligases were also identified. Related DNA ligases are encoded within several other phages, including: ΦLM21 (GenBank accession No. AII27824), *Rhizobium* phage vB_RleM_PPF1 (GenBank accession No. AID18355), and *Burkholderia* phage Bcepil02 (GenBank accession No. ACR15036), which may suggest their role in phage functioning, e.g., in recombination or integration of the virus, however this needs further analyses.

Moreover, in Φ2LM21 and Φ3LM21, besides genes encoding “typical” phage proteins, several additional modules (most probably comprising auxiliary metabolism genes) were identified. The regions carrying the extra genes are clustered within the right arm of the predicted prophage, downstream of the putative chitinase genes, which may indicate that they were hitchhiked from the bacterial hosts.

In Φ2LM21 the following functions for the “extra” genes were predicted: (i) *phi2LM21_p58* encodes a putative SOS response-associated peptidase (SRAP) of the SRAP family, which may act as a DNA-associated autoproteolytic switch that recruits diverse repair enzymes onto DNA damage [43]; and (ii) *phi2LM21_p68* encodes a putative nucleoid-associated NdpA-like protein exhibiting 52% identity to the appropriate protein of *Methylobacterium* sp. UNCCL125 (GenBank accession No. SFV08872). Moreover, within the right arm of the Φ2LM21 prophage, the abovementioned m^6^A MTase (Phi2LM21_p66) is also encoded.

In the Φ3LM21 prophage, the putative functions for four of the auxiliary metabolism genes were predicted. The *phi3LM21_p49* gene encodes an FkbM-like methyltransferase. It was shown previously that the homologs of this enzyme are required for specific methylation in the biosynthesis pathway of the macrocyclic polyketides (FK506 and FK520), with immunosuppressive activities in *Streptomyces* sp. strain MA6548 [44]. Interestingly, ΦLM21 also encodes a FkbM-like methyltransferase (GenBank accession No. AII27814), but both proteins seem to be unrelated. The *phi3LM21_p52*- encoded protein exhibited 72% identity to putative ammonia monooxygenase of *Rhizobiales* bacterium 68-8 (GenBank accession No. OJU35087). The ammonia monooxygenase is a metalloenzyme that catalyzes the oxidation of ammonia to hydroxylamine, which is the first step of nitrification of ammonia to nitrate [45]. Interestingly, the homologs of the Phi3LM21_p52 protein were found also in several phages, i.e., *Erwinia* phages, vB_EamM_Huxley and vB_EamM_ChrisDB, and *Ralstonia* phage, RSL2 (GenBank accession Nos. YP_009293074, YP_009292796, and YP_009213016). It was also revealed that the gene, *phi3LM21_p60*, encodes a putative ribose-phosphate pyrophosphokinase (Prs), showing the highest identity (99%) to related proteins in *Ensifer*/*Sinorhizobium* spp. (e.g., GenBank accession Nos. KDP75975, KQX04241, and KQZ45803). This enzyme transfers a pyrophosphoryl group from ATP to ribose 5-phosphate, synthesizing 5-phospho-α-d-ribose 1-diphosphate (PRPP). This reaction is needed during the synthesis of purines and pyrimidines, histidine and tryptophan amino acids, and NAD and NADP cofactors, and links these biosynthetic processes to the pentose phosphate pathway [46]. The last “additional” gene (*phi3LM21_p62*) of the Φ3LM21 prophage encodes a predicted lipopolysaccharide biosynthesis glycosyltransferase that may be involved in the addition of galactose or glucose residues to lipooligosaccharide (LOS) or lipopolysaccharide (LPS) of the bacterial cell surface [47]. Interestingly, genes encoding enzymes involved in LPS modification have been also identified in other temperate phages, e.g., phage ε15 conducting lysogenic conversion of *Salmonella enterica*, effecting in production of an altered form of LPS [48,49].

### 3.3. Characterization of the Φ4LM21 Prophage Remnant

The last prophage region, Φ4LM21, identified within the LM21 genome, seems to be incomplete and contains only structural and lysis genes (Figure 1, File S3). It was possible to distinguish 29 putative genes within this region and the potential function was assigned to 12 of them (Table S4). Amongst genes with predicted functions, there were eight encoding structural proteins: (i–ii) two baseplate assembly proteins J (genes *phi4LM21_p08–*p09); (iii) baseplate assembly protein W (*phi4LM21C_p10*); (iv) baseplate wedge component (*phi4LM21_p11*); (v) baseplate hub subunit and tail lysozyme (*phi4LM21_p12*); (vi) ATPase (*phi4LM21_p19*); (vii) tail tube protein (*phi4LM21_p23*); and (viii) tail sheath protein (*phi4LM21_p24*). Moreover, within the Φ4LM21, a putative gene (*phi4LM21_p03*) encoding the phage-related lysozyme (muraminidase) of GH24 family was identified. It exhibited the highest identity (99%) to several lytic enzymes of *Ensifer*/*Sinorhizobium* spp. (e.g., GenBank accession Nos. KQX25822, KSV67012, and SFH06584).

### 3.4. Functional Analyses of DNA Methyltransferases Encoded by the Sinorhizobium sp. LM21 Prophages

**Figure 2:**
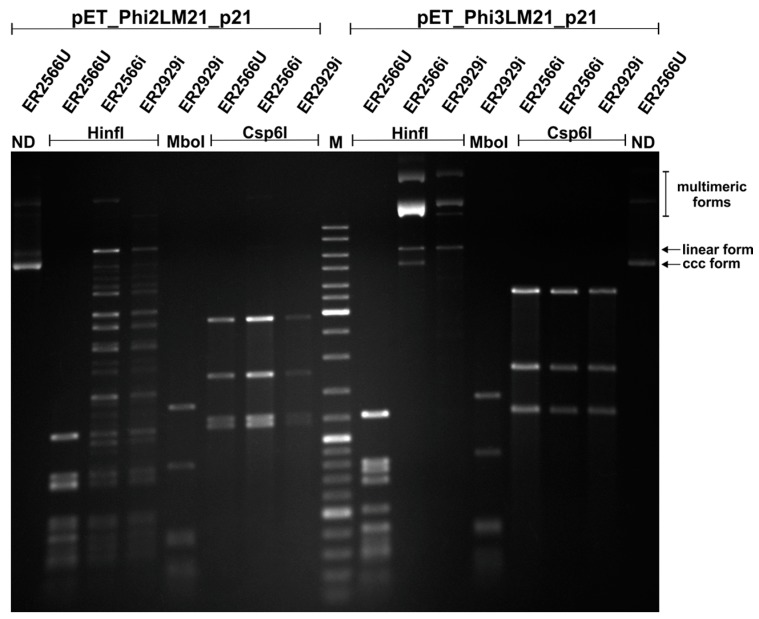
Comparative restriction patterns of the pET_Phi2LM21_p21 and pET_Phi3LM21_p21 plasmid DNAs prepared from *Escherichia coli* ER2566 or ER2929 cells grown in the presence (ER2566i or ER2929i, respectively) or absence (ER2566U) of inducer IPTG and cleaved with selected restriction endonucleases (*Hinf*I, *Mbo*I or *Csp6*I). Digest mixtures were electrophoresed on 0.8% agarose gel and stained with ethidium bromide. M—GeneRuler 100–10,000 bp size marker (Thermo Fisher Scientific, Waltham, MA, USA). The bands corresponding to supercoiled (ccc) and linear forms of the pET_Phi3LM21_p21 plasmid, as well as its multimeric forms were indicated.

As mentioned above, two genes, *phi2LM21_p21* and *phi3LM21_p21*, were predicted to encode m^6^A MTases. Protein products of these genes show 51% reciprocal identity, and, additionally, Phi2LM21_p21 and PhiLM21_p027 of ΦLM21 (GenBank accession No. AII27779) exhibit 33% identity. We demonstrated previously that the specificity of PhiLM21_p027 is GANTC (methylated nucleotide is underlined), the same as the host-encoded CcrM, a regulatory enzyme widespread among members of *Alphaproteobacteria*, although PhiLM21_p027 and CcrM_LM21_ do not share sequence similarities [14]. To determine whether GANTC sequences are substrates for Phi2LM21_p21 and Phi3LM21_p21, we digested the pET_Phi2LM21_p21 and pET_Phi3LM21_p21 plasmid DNAs isolated from IPTG-induced and non-induced *E. coli* cultures with *Hinf*I restriction enzyme (specificity GANTC, inhibited by m^6^A methylation). To confirm the susceptibility of the substrate DNA to digestion, a number of adenine methylation-sensitive and –insensitive endonucleases in an REase digestion assay were used. The DNAs isolated from the induced cultures were either fully (pET_Phi3LM21_p21) or partially (pET_Phi2LM21_p21) resistant to cleavage by *Hinf*I. All other REases were able to cleave substrate DNAs. Similarly, the pET_Phi2LM21_p21 and pET_Phi3LM21_p21 DNAs isolated from the non-induced cultures were susceptible to all restriction enzymes used, including *Hinf*I (Figure 2).

Additionally, in order to determine whether GATC sequences may also represent a substrate for Phi2LM21_p21 and Phi3LM21_p21 MTases, we re-transformed pET_Phi2LM21_p21 and pET_Phi3LM21_p21 plasmid DNAs to *E. coli* ER2929 Dam^−^ strain lysogenized with DE3 element (in DNAs isolated from the *E. coli* ER2566 all GATC sites are m^6^A modified due to the host EcoKDam MTase activity) [19]. The plasmid DNAs isolated from the induced cultures of *E. coli* ER2929 Dam^−^ strain were cleaved by MboI (GATC, inhibited by m^6^A methylation), while partial cleavage, with a large proportion of DNA fragments corresponding to linearized plasmids, was observed after using *Hinf*I (Figure 2).

Based on all obtained results, we concluded that the sequence specificity of both Phi2LM21_p21 and Phi3LM21_p21 was GANTC. Thereby, these enzymes were recognized as alphaproteobacterial phage MTases mimicking the sequence specificity of the host CcrM regulatory enzyme.

**Figure 3:**
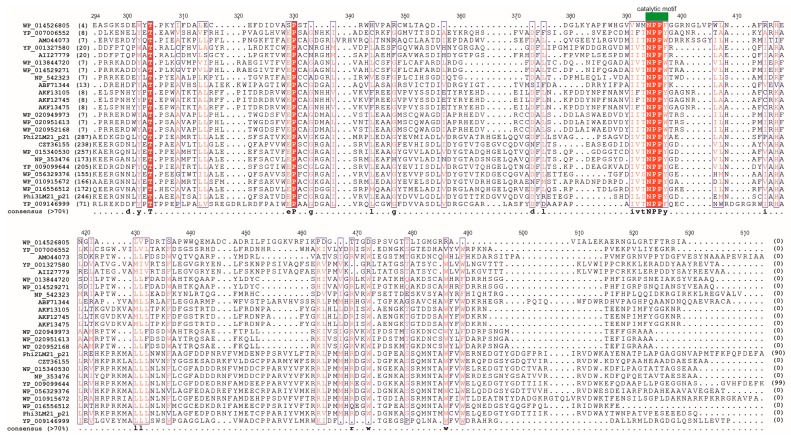
Alignment of the putative and experimentally confirmed CcrM-like specific DNA methyltransferases (MTases) found within the phages and predicted prophage regions of *Alphaproteobacteria*. The MTases with the following NCBI accession numbers were used for the alignment: AKF13105 (of ΦM19); AKF12745 (of ΦM7); AKF13475 (of ΦN3); YP_007006552 (of *Agrobacterium* phage 7-7-1); AMO44073 (of *Roseobacter* phage DSS3P8); YP_001327580 (of Φ3_WSM419); AII27779 (of ΦLM21); WP_013844720 (of Φ3_AK83); WP_014529271 (of Φ2_SM11); NP_542323 (of ΦPBC5); ABF71344 (of Φ16-3); WP_020949973, WP_020951613 and WP_020952168 (of *P. aminophilus* prophages); CZT36155 (of *Rhizobium* sp. 9140), WP_015340530 (of *Rhizobium tropici*); NP_353476 (of *Agrobacterium fabrum* str. C58); YP_009099644 (of *Rhizobium* phage vB_RleM_PPF1); WP_056329376 (of *Rhizobium* sp. Root482); WP_010915672 (of *Mesorhizobium loti*); WP_016556512 (of *Rhizobium grahamii*); and YP_009146999 (of *Aurantimonas* phage AmM-1). Additionally, within the alignment, MTases analyzed in this work (i.e., Phi2LM21_p21 and Phi3LM21_p21) were also included. The conserved amino acids were distinguished and/or presented within the consensus sequence. Moreover, catalytic motif (also known as motif IV of MTases) composed of NPP(Y/W/F) residues was indicated by a green block above the alignment. To retain transparency, the alignment was trimmed on both sides, and only its central, conserved region was presented. The numbers of trimmed amino acids have been provided in parentheses.

Previously, three GANTC-specific m^6^A MTases (JCM7686_1231, JCM7686_2255, and JCM7686_2934) were identified in prophage regions of the *Paracoccus aminophilus* JCM 7686 genome (one of these prophages, ФPam-6, turned out to be active) [5]. These small 179-amino acid proteins share putative catalytic (NPPW/F/Y) and *S*-adenosyl methionine (SAM)-binding motifs with the later-discovered 218-aa PhiLM21_p027 enzyme of ΦLM21 [14]. Interestingly, Phi2LM21_p21 and Phi3LM21_p21 proteins, both analyzed in this work, are much larger—599 and 456 aa, respectively. It should be stressed that genes encoding all the above mentioned GANTC-specific MTases are localized upstream of a cluster of genes presumably involved in phage replication. The same specificity of these MTase enzymes and the same localization of their genes within the phage genome strongly suggest relevance of methyltransferase activity for the phage replication. Noteworthy, we identified numerous homologs of these phage MTases with CcrM-like specificity in genomes of active, virulent (e.g., *Sinorhizobium* phage phiN3 (GenBank accession No. YP_009212452)) and temperate (e.g., *Rhizobium* phage vB_RleM_PPF1 (GenBank accession No. YP_009099644 of)) *Alphaproteobacteria* phages and even more of them within putative *Alphaproteobacteria* prophage sequences, which may suggest that this phenomenon is common in *Alphaproteobacteria* phages (Figure 3).

As these phage GANTC-specific MTases and CcrM proteins of their host are unrelated [14], it is therefore a clear example of evolutionary convergence of the sequence specificity of bacterial and phage CcrM-like enzymes in *Alphaproteobacteria*, similar to convergence of the GATC sequence specificity of bacterial and the majority of phage Dam-like proteins of *Gammaproteobacteria* [50].

Restriction enzyme digestion protection assay with panels of cytosine methylation-sensitive and adenine methylation-sensitive endonucleases were also used to test sequence specificity of Phi2LM21_p23, a putative m^5^C MTase, and Phi2LM21_p66, a putative m^6^A MTase. The DNAs of pET_Phi2LM21_p23 and pET_Phi2LM21_p66 isolated from the induced *E. coli* ER2566 cultures were sensitive to all restriction enzymes used in this test (data not shown), which suggests that the two remaining MTases of Φ2LM21 are inactive in a heterological host. In the case of Phi2LM21_p23, we can presume its specificity based on the similarity of this protein to JCM7686_0772 and JCM7686_2655—m^5^C MTases of *P. aminophilus* JCM7686 (44% identity), for which experimental data are available. They modify at least one cytosine in the CC motif [5]. Homologs of these relatively large (about 700 aa) phage proteins are widely distributed, not only in phage genomes of *Alpha*—(e.g., *Rhizobium* phage RR1-A) but also in *Gammaproteobacteria*. Similarly, m^5^C MTases with relaxed specificity are present in genomes of *Aeromonas* sp. ARM81 phages. Genes encoding ARM81mr_p29 of ФARM81mr and ARM81ld_p31 of the linear plasmid-prophage ФARM81ld (both have 34% identity with Phi2LM21_p23) are localized in a replication module (the same as Phi2LM21_p23) or in the vicinity of the plasmid partitioning system, respectively [51]. The location of these MTase genes adjacent to the replication/segregation module may suggest the relevance of the methyltransferase activity at this stage of the virus reproductive cycle.

### 3.5. Comparative Genomics and Networking of the Sinorhizobial (Pro)phages

For the comparative genomic analyses of the sinorhizobial viruses, 14 complete *Ensifer/Sinorhizobium* genomes available in GenBank (as of 1 March 2017) were screened for the presence of prophages. The use of the PhiSpy tool [23] indicated 46 potential prophage regions, which were afterwards verified by manual inspection (including identification of the predicted *att* sites). Finally, within the genomes of eight strains (i.e., *Ensifer adherens* Casida A (2 prophages), *Sinorhizobium medicae* WSM419 (3), *S. meliloti* RMO17 (1), *S. meliloti* AK83 (3), *S. meliloti* BL225C (2), *S. meliloti* Rm41 (2), *S. meliloti* SM11 (6), and *Sinorhizobium americanum* CFNEI73 (1)), 20 putative, complete prophages were identified and manually re-annotated (File S4).

Interestingly, until now, within the published data describing *Ensifer/Sinorhizobium* genomes, only a few prophage regions were mentioned (but not described in details) or the percentage contribution of the prophage regions within the particular bacterial genome was calculated [7,52]. This exemplifies the significant gap in our general knowledge concerning sinorhizobial (pro)phages. In this study, all (23) prophage regions identified within the sinorhizobial genomes, together with eight active lytic and temperate phages of *Sinorhizobium* spp. (i.e., ΦM12, ΦM7, ΦM19, ΦM9, ΦN3, Φ16-3, ΦPBC5, and ΦLM21) were subjected to thorough comparative analysis. The summary of the general features of particular sinorhizobial (pro)phages was presented in Table 1.

At first, all (predicted as complete) sinorhizobial (pro)phages compared in this work were subjected to analysis applying the VIRFAM tool [33], which enabled assigning of those viruses into appropriate families. It was revealed that within the analyzed pool of (pro)phages there were representatives of *Siphoviridae* (22 viruses), *Myoviridae* (6), and *Podoviridae* (3) (Table 1).

In the next step, with the application of the Circoletto tool [35], local nucleotide similarities within the genomes of analyzed (pro)phages were found (Figure S2). Although, all of the analyzed lytic phages were classified into T4 phage superfamily, the analysis confirmed previous findings showing that phages ΦN3, ΦM7, ΦM12, and ΦM19 create a separate group [not showing significant similarities with other sinorhizobial (pro)phages] and the ΦM9 was unique [12,53]. It is also worth mentioning that in 2016 phages ΦN3, ΦM7, ΦM12, and ΦM19 were clustered into a single genus called the M12-like viruses, and additionally phages ΦM12 and ΦM19 were considered as two strains of the same phage [53]. Furthermore, analyzing three others active, but temperate sinorhizobial phages (i.e., Φ16-3, ΦLM21, and ΦPBC5), we found that they show only partial (local) similarities to prophages identified within *Sinorhizobium/Ensifer* genomes and reciprocally (Figure S2).

**Figure 4:**
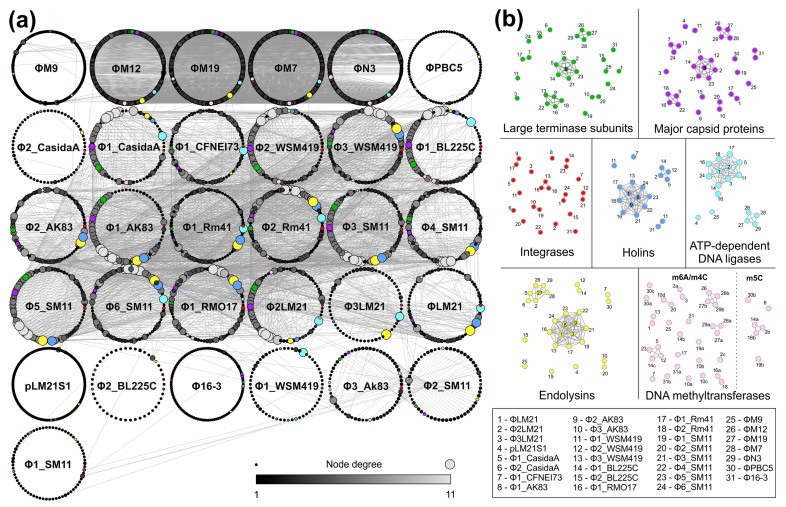
The similarity networks of the proteins encoded within the sinorhizobial phages. (**a**) The similarity network of 3688 sinorhizobial phages proteins. All the proteins (nodes) belonging to the same (pro)phage are circularly arranged and are linked to the others according to their identity value. The resulting picture for 50% threshold is shown. The size and color of each node (single protein) is proportional to its degree, which reflects the number of homologous proteins within the network (the more unique, the smaller and darker the node). Additionally, selected proteins were highlighted: large terminase subunits (green), major capsid proteins (magenta), integrases (red), holins (blue), ATP-dependent DNA ligases (light blue), endolysins (yellow) and DNA methyltransferases (pink). (**b**) Visualization of the similarity networks for selected proteins. The sequences of those proteins were presented as the multifasta files (File S5). Letters a, b and c beside the number of the (pro)phage indicate different DNA MTase encoded within the particular virus.

Following the general comparative analysis of the nucleotide sequences of sinorhizobial (pro)phages, all 3688 proteins encoded by 31 analyzed (pro)phages were used in all against all BLASTP searches (thresholds: 10^−5^ *e*-value, 50% identity and 50% of query coverage per subject) to construct protein similarity network. This resulted in a graph with 3688 nodes (proteins) and 3975 edges (reflecting reciprocal proteins similarities) which combined nodes into 666 subgraphs (groups of similar proteins) of different size and 1251 unique, one-element clusters (Figure 4). Amongst subgraphs, there were: 12s:6n, 11s:2n, 9s:5n, 8s:4n, 7s:26n, 6s:7n, 5s:26n, 4s:317n, 3s:98n, and 2s:175n, where s and n indicate the size (number of nodes) of a subgraph and the number of such subgraphs, respectively. This showed that 2437 (66.1%) of all analyzed proteins exhibited homology with at least one other protein in the dataset. Moreover, the analysis revealed that within the analyzed pool of (pro)phages there are highly unique ones, i.e., lytic phage ΦM9, temperate phages ΦPBC5, and Φ16-3, as well as predicted prophages Φ2_CasidaA, pLM21S1, Φ2_BL225C, Φ1_WSM419, and Φ1_SM11 (Figure 4). To allow transparent visualization of the selected protein networks, the separate clusterings were shown (Figure 4) and the sequences of those proteins were presented in the form of the multifasta files (File S5).

Firstly, three groups of proteins commonly used as phage phylogenetic markers [54] were subjected to analyses, i.e., large subunits of terminases (TerLs), integrases (Ints) and major capsid proteins.

The analysis of the large subunits of terminases revealed 11 unique proteins not showing significant similarity to other TerLs encoded by the sinorhizobial phages. Those were terminases encoded by ΦM9, ΦPBC5, Φ16-3, ΦLM21, pLM21S1, Φ3LM21, Φ1_SM11, Φ6_SM11, Φ1_WSM419, Φ2_BL225C, and Φ2_CasidaA. The analysis of the overall sinorhizobial phage proteins similarity network revealed that the remaining large subunits of terminases clustered into five multi-element groups, where four groups clustered exclusively TerLs of prophages distinguished in silico within the sinorhizobial genomes, while the last group gathered four TerLs of *Myoviridae* lytic phages (Figure 4).

The protein clustering showed that integrases for all (21) distinguished prophage sequences and three temperate active phages are highly diversified although they are all tyrosine-specific recombinases. The most numerous group gathered three Int proteins identified in Φ3_WSM419, Φ2_Rm21, and Φ1_RMO17, whereas the remaining integrases were clustered into five pairs and 11 unique proteins (Figure 4). The comparative analysis of the predicted attachment sites for identified prophages and the ΦLM21 phage revealed the strong congruence between the overall clustering of integrases and the nucleotide sequences of *attBs*, which may indicate the specificity of particular enzymes toward recognized DNA regions.

The analysis of the major capsid proteins revealed that they were clustered into six multi-element groups composed of: seven (one group), four (two groups), three (one group), and two (pairs) proteins. The remaining nine proteins were unique. The largest group was created by major capsid proteins of Φ1_CasidaA, Φ2_WSM419, Φ1_AK83, Φ1_BL225C, Φ3_SM11, Φ5_SM11, and Φ2LM21 (Figure 4).

In summary, following analysis of the similarity networks for three groups of proteins used as phage molecular markers we may conclude that large subunits of terminases (TerLs) and major capsid proteins represent congruent clustering, while integrases are much less conserved, and it would be difficult to use them as phylogenetic markers for temperate sinorhizobial viruses characterization.

The protein network analysis was also performed for other proteins encoded within examined (pro)phages. The analysis of the components of the phage lytic systems revealed the presence of 16 holins and 31 peptidoglycan hydrolases (endolysins), capable of degrading the bacterial cell wall. The analysis of the protein similarity networks revealed that distinguished holins were clustered into five groups comprised of: (i) 11 elements (predicted holins of ΦLM21, Φ3_WSM419, Φ1_AK83, Φ2_AK83, Φ1_Rm41, Φ2_Rm41, Φ1_RMO17, Φ3_SM11, Φ4_SM11, Φ5_SM11, and Φ6_SM11); (ii) four elements (LydA-like holins of Φ1_CasidaA, Φ1_WSM419, Φ1_BL225C, and Φ2LM21); (iii) two elements (holins of Φ1_WSM419 and Φ16-3); and (iv–v) one element (unique holins encoded by the phage Φ1_CFNEI73 and Φ1_WSM419) (Figure 4). Phage-encoded endolysins were clustered into five groups. The largest cluster consists of enzymes encoded by 11 prophages (Φ3LM21, Φ3_WSM419, Φ1_AK83, Φ2_AK83, Φ1_Rm41, Φ2_Rm41, Φ1_RMO17, Φ3_SM11, Φ4_SM11, Φ5_SM11, and Φ6_SM11) and a temperate phage ΦLM21. A seven-element cluster comprises predicted chitinases encoded by Φ1_CasidaA, Φ2_CasidaA, Φ2LM21, and four *Myoviridae* lytic phages. That was the first example when proteins encoded by the lytic phages did not cluster separately. There were also three pairs representing proteins most probably resembling different specificities, i.e., *N*-acetylmuramidases (of prophages Φ3_AK83 and Φ2_SM11), lambda-like lysozymes (of Φ1_WSM419 and Φ1_BL225C), and *N*-acetylmuramoyl-l-alanine amidases (of Φ1_CFNEI73 and ΦPBC5). Other distinguished endolysins were unique. Comparing the clustering of holins and endolysins, we found apparent congruency, which suggests that their genes form pairs and their products function as co-operating enzymes.

Within analyzed (pro)phages, 39 genes encoding DNA MTases were identified. The majority (33) of those enzymes were classified as m^6^A or m^4^C MTases. Only in seven prophages (pLM21S1, Φ1_WSM419, Φ1_AK83, Φ2_AK83, Φ2_BL225C, Φ4_SM11, and Φ6_SM11) were we not able to distinguish genes encoding DNA MTases. The highest number (4) of the MTase genes were identified in Φ3_AK83. In four (pro)phages (Φ1_BL225C, Φ2LM21, and ΦPBC5) as many as three genes encoding DNA MTases were identified, while, in six other (pro)phages (Φ1_RMO17, Φ1_SM11, Φ16-3, ΦM19, ΦM7, and ΦN3), two genes encoding DNA MTases were found. As shown by the protein network analyses, the identified DNA MTases are highly diverse, which make speculations about their DNA specificity difficult. On the other hand, it was noticed that most of the identified MTase genes were located in the proximity of the phage replication system, including all m^6^A MTases with an NPPY/F/W amino acid motif (the same as was previously shown for MTase genes of ΦLM21, Φ2LM21, and Φ3LM21). Therefore, we hypothesize that the identified MTases (with NPPY/F/W motifs) may also mimic the specificity of the host regulatory CcrM modifying enzyme (i.e., recognize and methylate GANTC sequence) and probably play a role in the virus cycle. The goal of future work is to determine the specific DNA sequences recognized by identified MTases of other sinorhizobial phages and test which of them exhibit CcrM-like specificity.

The analysis of the protein network revealed also that 17 (pro)phages encode ATP-dependent DNA ligases. Among these, 10 proteins encoded by prophages ΦLM21, Φ2LM21, Φ3LM21, Φ1_CasidaA, Φ1_CFNEI73, Φ1_WSM419, Φ1_BL225C, Φ1_Rm41, Φ2_Rm41, Φ1_RMO17, and Φ6_SM11 created a clustered group, in which the one encoded by Φ1_RMO17 seems to be most distinct. Another four-element cluster of ATP-dependent DNA ligases was created by those encoded by *Myoviridae* phages, which also encode homologous RNA ligases (data not shown). The last two ligases, identified in pLM21S1 and ΦM9, were unique.

Annotation of the phage genomes is still a challenging operation, as usually nearly 60–70% of genes remain annotated as encoding hypothetical proteins [55]. In this study, we faced the same problem, since 2667 (72.32%) of all analyzed (pro)phage proteins were initially annotated as hypothetical ones. After the manual re-annotation of the prophage regions identified within the *Sinorhizobium* spp. Genomes, we proposed the function for 141 (5.3%) predicted (previously hypothetical) proteins (File S4). Moreover, performing the large-scale protein networking analysis, we were able to suggest the possible function for the next 108 (4%) proteins, annotated previously as hypothetical. Those proteins in our analysis were clustered together with other proteins of predicted functions. Based on this result, we may conclude that the application of the complex manual annotation and high-throughput protein similarity network analysis in (pro)phage studies may significantly facilitate the future annotation of viral genomes and bring valuable suggestions concerning the possible function of the phage proteins for future experimental validations.

## 4. Conclusions

In the presented study, thorough manual analysis of the *Sinorhizobium* genomes revealed the presence of 23 prophages, which, together with eight previously identified active sinorhizobial phages, were subjected to complex comparative analyses applying protein networking. This study revealed that amongst analyzed viral proteins, holins, endolysins, and ATP-dependent DNA ligases are the most conserved, and it was shown that, especially, lytic enzymes form pairs whose genes are co-localized within particular phages. Moreover, congruence between the clustering of large subunits of terminases and major capsid proteins was observed, which reflects the phylogenetic relations between analyzed phages.

The analysis performed was the first such complex comparative study of the sinorhizobial phages. Using the example of *Sinorhizobium* phages, it was shown that application of complex manual annotation and high-throughput protein similarity network analysis may significantly improve overall phage annotation, as in this study we were able to suggest the possible function for nearly 10% of predicted proteins, previously annotated as hypothetical ones.

Moreover, in this study, it was shown that genes encoding DNA MTases are abundant in genomes of sinorhizobial phages and the phenomenon of the convergent evolution between phage MTases and the host regulatory CcrM MTase is common in *Sinorhizobium* spp., and most probably in other *Alphaproteobacteria*. Interestingly, it was also shown that the DNA MTases exhibiting CcrM-like specificity may not share high sequence similarity, however, they are all localized within the predicted replication modules of phages, which strongly suggests their regulatory role.

## Figures and Tables

**Table 1:** Summary of the general features of *Sinorhizobium*/*Ensifer* (pro)phages.

(Pro)phage Name	(Pro)phage Host	Host/Phage Accession Number	Coordinates in Host’s Genome	(Pro)phage Size (bp)	Number of Genes	Integration Site
Φ1_CasidaA	*E. adherens* Casida A	NZ_CP015880.1	2834796..2878945	44,150	62	tRNA-Leu (CAA)
		(chromosome)				
Φ2_CasidaA	*E. adherens* Casida A	NZ_CP015880.1	2972802..3012361	39,560	63	Intergenic region
		(chromosome)				
Φ1_CFNEI73	*S. americanum* CFNEI73	NZ_CP013107.1	1807693..1861334	53,642	84	tRNA-Cys(GCA)
		(chromosome)				
Φ1_WSM419	*S. medicae* WSM419	NC_009636.1 (chromosome)	1392222..1433999	41,778	58	tRNA-Ser(TGA)
Φ2_WSM419	*S. medicae* WSM419	NC_009636.1 (chromosome)	1717421..1768019	50,599	66	tRNA-dihydrouridine synthase A (DusA)
Φ3_WSM419	*S. medicae* WSM419	NC_009636.1 (chromosome)	1934112..1984910	50,799	72	tRNA-Lys(CTT)
Φ1_Rm41	*S. meliloti* Rm41	NC_018700.1 (chromosome)	742114..794018	53,565	80	tRNA-Ser(GCT)
Φ2_Rm41	*S. meliloti* Rm41	NC_018700.1 (chromosome)	1833694..1887258	51,921	86	tRNA-Lys(CTT)
Φ1_RMO17	*S. meliloti* RMO17	NZ_CP009144.1 (chromosome)	2233094..2285133	52,040	76	tRNA-Lys(CTT)
Φ1_BL225C	*S. meliloti* BL225C	NC_017322.1 (chromosome)	1366482..1418152	51,671	67	tRNA-Asn(GTT)
Φ2_BL225C	*S. meliloti* BL225C	NC_017323.1	1651701..1686916	35,216	46	tRNA-Arg(CCG)
		(pSINMEB02)				
Φ1_SM11	*S. meliloti* SM11	NC_017325.1 (chromosome)	912263..969433	57,171	69	tRNA-Met(CAT)
Φ2_SM11	*S. meliloti* SM11	NC_017325.1 (chromosome)	1084292..1130096	45,805	57	N/A
Φ3_SM11	*S. meliloti* SM11	NC_017325.1 (chromosome)	1453058..1501481	48,424	63	tRNA-dihydrouridine synthase A (DusA)
Φ4_SM11	*S. meliloti* SM11	NC_017325.1 (chromosome)	1795391..1849554	54,164	81	tRNA-Leu(TAA)
Φ5_SM11	*S. meliloti* SM11	NC_017325.1 (chromosome)	1864579..1915967	51,389	72	tRNA-Asn(GTT)
Φ6_SM11	*S. meliloti* SM11	NC_017325.1 (chromosome)	2351730..2402613	50,865	76	tRNA-Pro(GGG)
Φ1_AK83	*S. meliloti* AK83	NC_015590.1 (chromosome 1)	264329..313309	48,981	62	tRNA-Thr(GGT)
Φ2_AK83	*S. meliloti* AK83	NC_015590.1 (chromosome 1)	795050..847939	52,890	78	tRNA-Ser(GCT)
Φ3_AK83	*S. meliloti* AK83	NC_015590.1 (chromosome 1)	2309762..2355282	45,521	61	tRNA-Met(CAT)
pS1LM21	*Sinorhizobium* sp. LM21	KM659098	N/A	117,539	150	N/A
ΦLM21	*Sinorhizobium* sp. LM21	KJ743987	N/A	50,827	72	tRNA-Pro(GGG)
Φ2LM21	*Sinorhizobium* sp. LM21	SAMN06765771	550879..597478 (Contig_2)	46,599	69	tRNA-Phe(GAA)
Φ3LM21	*Sinorhizobium* sp. LM21	SAMN06765771	78163..119610 (Contig_16)	41,447	59	tRNA-Pro(CGG)
Φ16-3	*S. meliloti* Rm41	DQ500118	N/A	60,195	110	tRNA-Pro(CGG)
ΦPBC5	*S. meliloti* 2011	NC_003324	N/A	57,416	83	N/A
ΦM7	*S. meliloti* (lh) ^1^	KR052480	virulent	188,427	359	N/A
ΦM9	*S. meliloti* (lh)	KP881232	virulent	149,218	271	N/A
ΦM12	*S. meliloti* (lh)	KF381361	virulent	194,701	377	N/A
ΦM19	*S. meliloti* (lh)	KR052481	virulent	188,047	361	N/A
ΦN3	*S. meliloti* (lh)	KR052482	virulent	206,713	398	N/A

^1^ lh—laboratory host (the strain used for identification of the phage), bp: base pair.

## Supplementary Materials

The following are available online at www.mdpi.com/1999-4915/9/7/161/s1, Figure S1: Profiles of *E. coli* ER2566 cell lysis as the result of Phi2LM21_p54 expression; Figure S2: Comparative genomic analyses of 31 sinorhizobial (pro)phages; Table S1: Oligonucleotide primers used in this study; Table S2: Genes located within the Φ2LM21 prophage; Table S3: Genes located within the ΦL3M21 prophage; Table S4: Genes located within the Φ4LM21 prophage remnant; File S1: GenBank file with annotated sequence of the Φ2LM21 prophage; File S2: GenBank file with annotated sequence of the Φ3LM21 prophage; File S3: GenBank file with annotated sequence of the Φ4LM21 prophage remnant; File S4: Combined GenBank files with annotated sequences of 20 putative, complete prophages retrieved from the *Ensifer/Sinorhizobium* genomes; File S5: Combined multifasta files with amino acid sequences of the large subunits of terminases, major capsid proteins, integrases, holins, endolysins, ATP-dependent DNA ligases and DNA methyltransferases of analyzed *Ensifer/Sinorhizobium* (pro)phages.
